# Supporting open, collaborative, evidence-based scholarship: considering the future of perspectives on medical education

**DOI:** 10.1007/s40037-021-00697-2

**Published:** 2021-11-24

**Authors:** Erik Driessen, Lauren A. Maggio, Teresa Chan, Martin Tolsgaard, Kal Winston

**Affiliations:** 1 Department of Educational Development and Research, Maastricht University, Maastricht, The Netherlands; 2 Department of Medicine, Uniformed Services University of the Health Sciences, Bethesda, MD USA; 3 Department of Medicine, McMaster University, Hamilton, Ontario Canada; 4 Rigshospitalet, Copenhagen Academy for Medical Education and Simulation (CAMES), Copenhagen, Denmark; 5 Department of Public Health and Primary Care, University of Cambridge, Cambridge, UK

*‘In my opinion, it is essential that Perspectives achieves an impact factor, as this is likely to entice significantly more researchers to opt for Perspectives as the platform for their publications.’* I (Erik) wrote these words in my first editorial published in January 2014 [1]. Seven years later, we are proud to announce that we have fulfilled this ambition, among several others.

In 2021, *Perspectives on Medical Education* (PME) was selected for Web of Science and assigned an impact factor of 2.974 (2021). We thank our many readers, (including teachers, researchers, educational developers and policy makers), and authors who have found their way to the journal for this achievement. Article downloads have increased from 13,472 (2013) to 474,632 (2020) and manuscript submissions from 178 (2013) to 842 (2020). This increased visibility may be both a cause and consequence of the journal’s selection not only for Web of Science, but also MEDLINE, Scopus, and the Directory of Open Access Journals (DOAJ). We also have our own Wikipedia page (https://en.wikipedia.org/wiki/Perspectives_on_Medical_Education) and several PME articles have been featured on the KeyLiME podcast [2].

While we are thrilled with this great success, it also brings greater responsibility. PME must continue to serve the field by empowering readers and authors, and must do so using a sustainable journal model. In this editorial, we outline our plans for the coming years.

Our plans focus on collaborative scholarship between education researchers and educators, championing open science, taking an evidence-informed approach to running PME, and supporting the development of junior scholars.

## Collaborative scholarship

PME will continue supporting scholarly conversations on the cutting edge of educational research and clinical education. The journal’s mission is to support and enrich collaborative scholarship between education researchers and health professions educators, and to advance new knowledge regarding the education practices of clinical health professions. We wish to place PME within the overlapping interest areas for scientists and educators, also known as Pasteur’s quadrant [3]. Balancing between ivory tower research and ‘in the trenches’ studies requires a broad scope [4]. PME’s scope includes papers practically oriented (Edison’s quadrant) and those that are purely theoretical (Bohr’s quadrant). This interplay between research paradigms, ambitions and scopes is crucial for the scientific credibility, educational impact and thereby the journal’s sustainability. We, therefore, publish a variety of papers and publication types. For example, Guidelines articles support busy educators with their teaching (e.g. Guidelines: The dos, don’ts and don’t knows of remediation in medical education [5]). We also promote excellent writing with the Writers’ Craft series (e.g. Collaborative writing: Strategies and activities for writing productively together [6]). With the Insiders’ Perspectives papers, we support the development of new members in our field (e.g., Islands and archipelagos: Reconciling programmatic vs. opportunistic research in health professions education [7]).

We are committed to contributing to knowledge translation and dissemination of research insights through publishing concise papers written in clear language. Health professions education researchers often use complex research methods, and complex and abstract theories. Our goal is to ensure these studies are reported in articles that are approachable for every interested teacher willing to look up a few words in a dictionary. We have an ambition to move beyond traditional PDF papers and adopt new forms of scholarly engagement as they emerge by capitalizing on the affordances that current technology provides.

## Championing open science

PME will continue to fully support open science. We are a non-profit open access journal generously supported by the Netherlands Association of Medical Education. The journal is free of charge for both authors and readers, and authors retain their article’s copyright. We believe that being open access has greatly contributed to PME’s popularity. However, as we continue to grow, we must continue to investigate sustainable publishing models to ensure a robust future for PME. For example, we must ensure that we are able to handle our increasing popularity with an eye towards potentially expanding the number of articles we can publish. To this end, we are exploring alternate models to understand how other open access journals have navigated these challenges.

Beyond open access, we encourage authors to deposit preprints. See our editorial on preprints for more information on this topic [8]. Additionally, we encourage data deposit and submission of replication studies.

## Taking an evidence-informed approach

We believe that an evidence-based journal approach can support the development of both PME and our field. Therefore, we aim to critically examine PME by conducting meta-research both internally (e.g., evaluating PME’s review and editorial processes) and externally (e.g., understanding how our articles are used). Thus, in alignment with journals such as *BMJ* and *PLOS*, we have instituted a policy to study PME. In 2022, we will publish a special issue on meta-research [9].

## Empowering junior researchers

We want to support junior scholarship not only through publishing papers, but also by offering internships. We believe that fostering the field of health professions education begins by providing opportunities for junior scholars to engage in apprenticeship and on-the-job learning as members of our editorial board. We will publish the details of this new initiative in 2022.

With these plans, we strive to make Perspectives an exciting and thought-provoking journal that caters to the interests of all those involved in healthcare education.
